# Parallel Import: Is It Worth?

**Published:** 2014

**Authors:** Farzad Peiravian


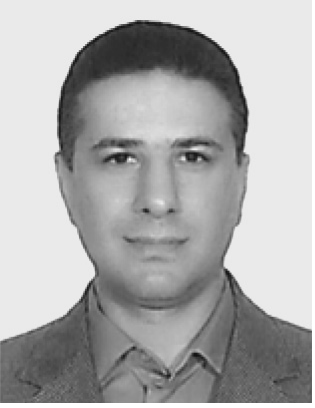


One of the most important duties of government in public health sector is to provide secured sources for medicines supply. There are some regulations for domestic pharmaceutical industry. In addition, there is a discipline for importing medicine to be ensured about their claimed characteristics. In medicines registration process, MOH (FDO in Iran) assess ‘safety’, ‘quality’ and ‘efficacy’ of medicines and they will be registered if they’ll be able to pass the assessment. Some countries establish ‘cost- effectiveness’ as fourth hurdle in registration process of medicines. It means that at first authorities must be ensured about safety, quality and efficacy of medicines.

In Iran, there are two main importing forms of medicines. One is by agents, who registered the medicines and have responsibility for them. Agents should trace medicines from buying stage to shipment, delivering and distributing by wholesalers and selling by pharmacies. In addition, they are responsible for safety, quality and efficacy and pharmacovigilance of medicines. Two is by importers (Emergency companies). The main activity of them is supply those medicines which FDO announced as shortages. They could import medicines from registered wholesalers in confirmed countries based on its specific regulation. In this form of import, FDO rely on confirmed countries’ regulations to ensure about safety, quality and efficacy of required medicines.

On the other hand, people ability to pay and financial constrain push governments to focus on the ways which help them to supply drugs in lowest price with respect to those hurdles. Pricing is one of the tools that FDO use in order to decrease and control cost of medicines. Mechanism of pricing in domestic medicines is based on cost plus method and in importing medicines is external reference based pricing. Turkey, Greece and Spain are the main countries as medicines reference price in Iran. Generally, registered branded medicine have imported exclusively by specific agents. Because of patent protection rules of branded medicines during their patent period in the world, they have a kind of market power. Level of market power of these medicines is depends on market structure, demand elasticity, pricing regulations and competition policies. These factors give a kind of monopoly to branded medicines in Iran and consequently, agents tend to set their branded medicines price as much higher as they can. 

In most cases, branded medicines originators charge lower prices for a medicine in one country than in another, taking into account their market power. This means that a country with limited resources can sometimes afford more of patented medicines by purchasing them in other countries at a lower price and importing them, rather than buying them directly in its domestic market at the higher price. Most of the time in Iran, the price of these medicines is higher than reference price countries. Many countries’ patent laws dictate that once a patent owner sells its goods in any country, it has no right to control the resale of those goods (so-called “regime of international exhaustion”). In legal terms, the patent owner has “exhausted” its property rights in the product actually sold while it maintains the exclusive right to manufacture the product, but it cannot use its intellectual property rights to prevent resale of those products it sells. Thus importers could buy a patented medicine in one country at the lower price and then resell the medicine in another country at a price that is higher but still undercuts what the manufacturer is charging for its patented medicine in that country by their agents. This is called “parallel importing”.

Parallel import (PI) has been used in several countries such as EU. In 1990, PI devoted perhaps two percent market share of the prescription drug market in the European Countries overall, with ranging from one percent in Germany to 5-10 percent in the Netherlands and eight percent in the UK .

Most of the EU countries have some requirements for parallel importing of medicinal products. For example imported product has to be granted a marketing authorization in the Member State of Origin, product is essentially similar to a product that has already received marketing authorization in the Member State of destination, importer has to have PI license, manufacture’s authorization for the company responsible for re-labeling, re-packaging and re-call issued by the regulatory authority, batch control and batch testing and wholesalers authorization. Obviously parallel import has occurred in EU between European countries with approximately similar regulations and supportive agreement in this field. 

There are some arguments about PI. Opponents of PI often claim that PI would support consumer deception and trade in counterfeit goods and pirated goods. 

The benefits of parallel imports are:

1. Direct reduction in branded medicines price.

2. PI can be a complement to price control strategies. It provides health providers strong negotiating leverage with original manufacturers that they cut their prices down. 

The costs of parallel imports are:

1. To the extent that original manufacturers set prices according to factors influencing market power, integrating markets through PI could raise the prices in exporting countries by reducing available medicines in supply chain. In this condition, firms could refuse to supply those markets.

2. Costs of transportation and repackaging in parallel trade may decrease a significant portion of any potential price advantages.

3. PI firms have no costs in R&D and marketing. They normally use marketing expenses of original manufacturers and their licensees, which could reduce their willingness to supply certain markets and products.

4. PI reduces profitability of original manufacturers. It can affect their R&D programs that are sensitive to such profit reductions and it can lead to slow down global drug development.

Parallel import is one of the policies that FDO adopt to cut the medicines price these days. It means that FDO allows the importers to (PI) some branded medicines. This policy has some pros and cons in Iran. Unfortunately, high amount of medicines that have been imported by importers (not agents) are coming from unregulated countries. It means that importers buy medicines from wholesalers in other countries that are not regulated from FDO’s point of view. It makes us worried about safety, quality and efficacy of these medicines. PI in this shape won’t be able to guarantee these requirements. It’s possible to counterfeit medicines are parallel imported to the country via countries of beneficiary’s unregulated market. 

Counterfeit medicines are deliberately and fraudulently mislabeled with respect to identity and/or source. Counterfeiting can be relevant to both branded and generic medicines. Counterfeit medicines may include products with the correct ingredients but fake packaging, without active ingredients, with insufficient active ingredients or with the wrong ingredients. In 2003, the World Health Organization cited estimates that the annual earnings from substandard and/or counterfeit drugs were over US$32 billion.

 In addition because there is not enough supervision on route of transportation of medicines in PI (for example those which need cool chain for transportation), medicines might be corrupted and become out of use before their expiry date. Since branded owners don’t accept the responsibility of parallel imported medicines in Iran, responsibility for adverse reactions of these medicines is unclear. In addition, according to mechanism of medicines supply in PI, sustainability in supply of parallel-distributed medicines is ambiguous. At last, current laboratories capacity in Iran is not enough to detect all kinds of substandard and counterfeiting medicines.

It’s not logical to prioritize price and affordability over safety, quality and efficacy. FDO’s supposed to find the most affordable medicines within those with acceptable safety, quality and efficacy. PI in Iran should have a role as supporting tool for imported medicines’ supply, not the main tool.

There are two suggestions in order to reach to acceptable price in compare with reference price countries. 

1- Negotiation with agents and branded owners in order to cut their medicines price down at the logical level in compare with reference price countries. It’s obvious that small PI via trustable route can help to find real price level in other countries.

2- In all possible cases, ‘me-too’ medicines can act as a strong competitor to branded medicines. There are so many branded- generic medicines that have similar effect and indications with acceptable requirements. Formal importing of these medicines can push branded owners to cut their price down to maintain and support their market share. Totally, it seems PI in Iran has some advantages in the short-run but in the long- run, that’s not guaranteed. 

*Farzad Peiravian is currently working as assistant Professor of Department of Pharmacoeconomic and Phrma Management, School of Pharmacy, Shahid Beheshti University of Medical Sciences, Tehran, Iran. He could be reached at the following e-mail address: peiravianfarzad@gmail.com*

